# Expression of the Circadian Clock Gene *BMAL1* Positively Correlates With Antitumor Immunity and Patient Survival in Metastatic Melanoma

**DOI:** 10.3389/fonc.2018.00185

**Published:** 2018-06-12

**Authors:** Leonardo Vinícius Monteiro de Assis, Gabriela Sarti Kinker, Maria Nathália Moraes, Regina P. Markus, Pedro Augusto Fernandes, Ana Maria de Lauro Castrucci

**Affiliations:** ^1^Laboratory of Comparative Physiology of Pigmentation, Department of Physiology, Institute of Biosciences, University of São Paulo, São Paulo, Brazil; ^2^Laboratory of Neuroimmunemodulation, Department of Physiology, Institute of Biosciences, University of São Paulo, São Paulo, Brazil; ^3^Department of Biology, University of Virginia, Charlottesville, VA, United States

**Keywords:** skin cancer, melanoma, circadian rhythms, clock genes, ARNTL/BMAL1 immunotherapy

## Abstract

**Introduction:**

Melanoma is the most lethal type of skin cancer, with increasing incidence and mortality rates worldwide. Multiple studies have demonstrated a link between cancer development/progression and circadian disruption; however, the complex role of tumor-autonomous molecular clocks remains poorly understood. With that in mind, we investigated the pathophysiological relevance of clock genes expression in metastatic melanoma.

**Methods:**

We analyzed gene expression, somatic mutation, and clinical data from 340 metastatic melanomas from The Cancer Genome Atlas, as well as gene expression data from 234 normal skin samples from genotype-tissue expression. Findings were confirmed in independent datasets.

**Results:**

In melanomas, the expression of most clock genes was remarkably reduced and displayed a disrupted pattern of co-expression compared to the normal skins, indicating a dysfunctional circadian clock. Importantly, we demonstrate that the expression of the clock gene aryl hydrocarbon receptor nuclear translocator-like protein 1 (*BMAL1*) positively correlates with patient overall survival and with the expression of T-cell activity and exhaustion markers in the tumor bulk. Accordingly, high *BMAL1* expression in pretreatment samples was significantly associated with clinical benefit from immune checkpoint inhibitors. The robust intratumoral T-cell infiltration/activation observed in patients with high *BMAL1* expression was associated with a decreased expression of key DNA-repair enzymes, and with an increased mutational/neoantigen load.

**Conclusion:**

Overall, our data corroborate previous reports regarding the impact of *BMAL1* expression on the cellular DNA-repair capacity and indicate that alterations in the tumor-autonomous molecular clock could influence the cellular composition of the surrounding microenvironment. Moreover, we revealed the potential of *BMAL1* as a clinically relevant prognostic factor and biomarker for T-cell-based immunotherapies.

## Significance

Here, we provide a first glimpse regarding the impact of a disrupted tumor-autonomous molecular clock on the cellular composition of the tumor microenvironment through the modulation of DNA-repair capacity. Within this line, our data revealed the potential of *BMAL1* as a clinically relevant biomarker for immunotherapy response and overall survival of patients with metastatic melanoma.

## Introduction

Melanoma is the most lethal type of skin cancer, with increasing incidence and mortality rates worldwide (1, 2). It represents only 4% of skin cancer but accounts for approximately 80% of skin cancer-related death (3). Although complete surgical resection is often curative for melanomas detected at initial stages, patients with metastatic disease have an overall survival of approximately 5 months (4). Therapeutic options for patients with metastatic melanoma have dramatically changed in the past years, with the introduction of more effective agents such as proto-oncogene, serine/theronine kinase (BRAF), mitogen activated protein kinase kinase (MAPK), and immunotherapeutic antibodies directed to cytotoxic T-lymphocyte-associated antigen 4 (CTLA-4), programmed cell-death protein 1 (PD-1) and its ligand (PD-L1) (5–8). Melanoma etiology is multifactorial and includes risk factors such as ultraviolet radiation exposure, genetic susceptibility, high nevus density, reduced skin pigmentation, and immunosuppression (9, 10).

Proper temporal control of physiological functions is crucial for maintaining the homeostasis of multi-cellular organisms (11–13). In mammals, the molecular machinery of timekeeping and circadian rhythm generation is based on interconnected positive and negative transcriptional–translational feedback loops. The central hypothalamic clock (suprachiasmatic nuclei, SCN) and clocks located in peripheral tissues share the same molecular architecture, engaging core genes such as aryl hydrocarbon receptor nuclear translocator-like protein 1 (*BMAL1* also known as *ARNTL*), cryptochrome 1 and 2 (*CRY1/2*), circadian locomotor output cycles kaput (*CLOCK*), period 1, 2, and 3 (*PER1/2/3*), receptor subfamily 1, group D, member 1/2 (*NRD1/2* also known as *REV-ERB*α*/*β), and RAR-related orphan receptor A and B (*RORA/B* also known as NR1F1/2). In healthy conditions, CLOCK–BMAL1 heterodimers translocate to the nucleus and induce the gene expression of their own inhibitors, PER and CRY proteins. This core oscillatory pathway is augmented and stabilized by a secondary loop involving *NRD1/2* and *RORA/B*, nuclear receptors that modulate *BMAL1* expression. Importantly, CLOCK–BMAL1 heterodimers also regulate the expression of several clock-controlled genes, which are tissue- and cell type-specific (11–13).

Many epidemiologic studies have demonstrated that the disturbance of biological rhythms through shift work, increased light exposure at night, and irregular feeding regimens (14–16) is associated with increased risk of developing several types of cancers (17–19). In fact, alterations in the cellular circadian machinery have been shown to affect cancer-related processes such as cell proliferation (20, 21), DNA damage response (22, 23), and metabolism (24–27) in a tumor-specific manner. Accordingly, the aberrant expression of clock core genes such as *CRY1, PER1*, and *PER2* has been shown to impact tumor progression in colorectal, prostate, and breast cancers, respectively (28–30).

In melanoma, mRNA levels and nuclear immunopositivity for CLOCK, CRY1, and PER1 are reduced compared to adjacent non-tumorous skin and present a significant association with clinicopathological features such as Breslow thickness (31). Moreover, the expression of RORA is lower in melanomas than in nevi, and positively correlates with overall survival and disease-free survival (32). Interestingly, enhancing the circadian clock function of melanoma cells impairs cell cycle progression and inhibits tumor growth *in vivo* (21). In this sense, we have previously demonstrated that the expression of clock core genes in murine melanoma cells can be activated by different stimulus, such as white light exposure (33), UVA radiation (34), estradiol (35), and thermal energy (36). Recently, we have demonstrated that a non-metastatic model of melanoma leads to a systemic chronodisruption in tumor-adjacent skin, lungs, liver, and SCN, as in these tissues the rhythmic expression of *Bmal1* was lost in tumor-bearing mice (37). These data reinforce that the modulation of tumor-autonomous clock might represent a novel and promising therapeutic strategy.

To further characterize the pathophysiological relevance of the molecular clock in skin cancer, we investigated the clinical value of clock core genes expression in metastatic melanoma, using public high-throughput molecular data. Overall, we revealed the robust prognostic power of *BMAL1* expression and provided evidence into its underlying biological processes.

## Materials and Methods

### Datasets of Melanoma and Normal Skin Samples

Gene expression, somatic mutation, and clinical data from 340 metastatic melanomas from The Cancer Genome Atlas (TCGA) and gene expression data from 234 Genotype-Tissue Expression (GTEx) normal skin (not sun exposed) samples were downloaded from the UCSC XENA Browser (http://xena.ucsc.edu) in January of 2017. TCGA and GTEx gene expression data were originally generated by TCGA (38) and GTEx consortia (39), respectively, using the Illumina HiSeq 2000 RNA sequencing platform, quantified using RSEM, upper quartile normalized and log_2_(*x* + 1) transformed. TCGA somatic mutation data were generated using the Illumina GAIIx DNA sequencing platform and somatic variants (SNPs and small indels) were identified using MuTect2. Neoantigen load information for TCGA metastatic melanoma samples was obtained from Rooney et al. (40). Briefly, for each metastatic melanoma patient, all novel amino acid 9–10mers resulting from missense mutations in expressed genes (median > 10 TPM) were identified. Mutant peptides with a HLA-binding affinity <500 nM, predicted by NetMHCpan (v2.4), were considered antigenic (41). Clinical information and gene expression data of pretreatment biopsies from 49 patients who received anti-PD1 immunotherapy (nivolumab) were obtained from Riaz et al. (42). Expression data were generated using the Illumina HiSeq 2000 RNA sequencing platform, counted using Rsamtools v3.2, upper quartile normalized and log_2_(*x* + 1) transformed. Treatment response for patients was defined by RECIST v1.1.

### Co-Expression Network Analysis

Undirected weighted co-expression networks were constructed based on the pairwise Spearman’s correlation coefficients between the expression of clock core genes *BMAL1, CRY1, CRY2, NRD1, PER1, PER2, PER3*, and *RORA*. Using the CoGA R package (43), we compared the structural properties of co-expression networks from normal skin and metastatic melanomas by testing the equality in their spectral distributions (44, 45). The spectrum of a graph, defined as the set of eigenvalues of its adjacency matrix, describes several structural features and represents a comprehensive characterization of networks (44, 46). *P*-values were calculated based on 1,000 phenotype permutations and networks were visualized using the gplots R package.

### Gene Set Enrichment Analysis (GSEA)

Genes in the TCGA expression dataset were ranked according to the Spearman’s correlation coefficient between their expression and the expression of *BMAL1*. GSEA was performed using GSEA v3.0 and Reactome pathways (47, 48). Enrichment scores (ES) were calculated based on a weighted Kolmogorov–Smirnov-like statistic and normalized (NES) to account for the size of each gene set. *P*-values corresponding to each NES were calculated based on 1,000 phenotype permutations and corrected for multiple comparisons using the false discovery rate (FDR) procedure. Adjusted *P*-values < 0.05 were considered statistically significant.

### Single Sample Gene Set Enrichment Analysis (ssGSEA)

Single sample gene set enrichment analysis, an extension of GSEA, was used to estimate the degree of enrichment of gene sets in individual samples within the TCGA gene expression dataset (49). For each sample, gene expression values were rank-normalized, and ESs were calculated based on the difference between weighted Empirical Cumulative Distribution Functions of genes inside and outside the gene sets. We performed ssGSEA using the GSVA R package (50) and DNA repair-related KEGG pathways (51), namely: base excision repair (hsa03410), nucleotide excision repair (hsa03420), mismatch repair (hsa03430), homologous recombination (hsa03440), and non-homologous end joining (hsa0345).

### Statistical Analysis

We used the two-sided Wilcoxon–Mann–Whitney test to perform two-group comparisons, the Spearman’s correlation test to assess ordinal associations, and the Chi-square test to analyze the relationship between two categorical variables. The impact of clock core genes expression on patient overall survival was evaluated using univariate Cox regressions. The prognostic power of *BMAL1* expression was further investigated using Kaplan–Meier curves, combined to the log-rank test, and multivariate Cox regressions. Hazard Ratios, including 95% confidence intervals, were calculated. Statistical analyses were performed with GraphPad Prism 6 and R (www.r-project.org). *P*-values < 0.05 were considered statistically significant. Where indicated, *P*-values were adjusted for multiple comparisons using the FDR procedure.

## Results

### Clinical Relevance of Clock Core Genes Expression in Metastatic Melanomas

**Figure 1:**
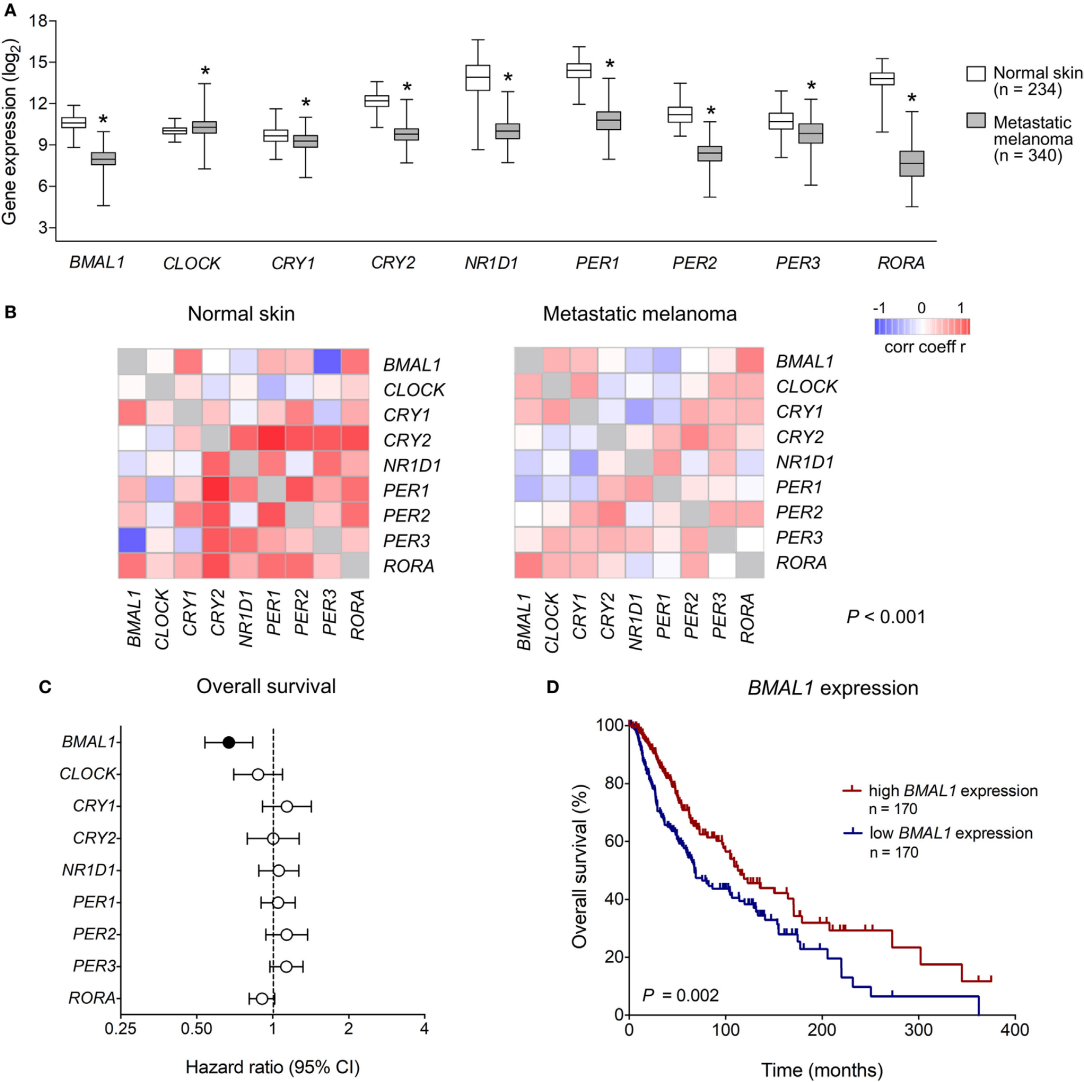
Clinical relevance of clock genes expression in metastatic melanomas. **(A)** RNAseq analysis of clock genes expression in genotype-tissue expression (GTEx) normal skins (*n* = 234) and The Cancer Genome Atlas (TCGA) metastatic melanoma (*n* = 340). Expression values were estimated using RSEM and log_2_(*x* + 1) transformed. The boxes extend from the 25th to the 75th percentile, the central bold line shows the median, and whiskers are drawn from minimum to maximum values. Comparisons were performed using the two-sided Wilcoxon–Mann–Whitney test. *Significantly different from normal skin (*P* < 0.05). **(B)** Co-expression matrix showing pairwise Spearman’s correlation coefficients of clock core genes in GTEx normal skins (*n* = 234) and TCGA metastatic melanomas (*n* = 340). Networks were compared using the CoGA software. **(C)** Univariate Cox analysis of overall survival according to the expression of clock core genes in TCGA metastatic melanomas. Hazard Ratios including 95% confidence intervals are shown. Genes with a significant prognostic value (*P* < 0.05) are marked in black. **(D)** Kaplan–Meier survival curve according to the expression of aryl hydrocarbon receptor nuclear translocator-like protein 1 (*BMAL1*) in TCGA metastatic melanomas. The median expression of *BMAL1* was used as the cutoff to dichotomize the population. Comparisons were performed using the log-rank test.

We first analyzed the expression of clock core genes in normal skin and in metastatic melanomas. Compared to normal skin, metastatic melanomas demonstrated a remarkably decreased expression of *BMAL1, CRY1, CRY2, NRD1, PER1, PER2, PER3*, and *RORA* and an increased expression of *CLOCK* (Figure 1A). In normal skin, we have found a classic pattern of clock gene expression: *PER*s and CRYs are concomitantly expressed (in phase) and are in antiphase with *BMAL1* and *CLOCK* expression, as expected; on the other hand, in metastatic melanomas such correlations are severely attenuated (Figure 1B), which further corroborates a dysfunctional circadian clock within the tumor. In metastatic melanomas, male presented increased percentage of tumor showing high expression of *NRD1, PER2*, and *PER3* (*P* = 0.015, *P* = 0.028, and *P* < 0.001, respectively; Table 1; Table S1 in Supplementary Material). Patients with high *PER3* expression were also significantly older and more frequently diagnosed with stage I–II tumors (*P* = 0.002 and *P* = 0.037, respectively; Table 1; Table S1 in Supplementary Material).

**Table 1:** Clinicopathological features according to the expression of clock genes in The Cancer Genome Atlas metastatic melanomas.

	*P*-values*								
Variables	*BMAL1*	*CRY1*	*CRY2*	*CLOCK*	*NR1D1*	*PER1*	*PER2*	*PER3*	*RORA*
Age	0.59	0.592	0.998	0.151	0.057	0.19	0.754	**0.002**	0.286
Gender	0.659	0.271	0.269	0.269	**0.015**	0.06	**0.028**	**<0.001**	0.269
Pathologic stage	0.817	0.643	0.644	0.083	0.247	0.418	0.132	**0.037**	0.417
Ulceration status	1	0.404	0.094	0.889	1	0.78	0.267	0.889	1
Mitotic count	0.769	0.175	0.07	0.801	0.465	0.256	0.276	0.613	0.963
Breslow thickness	0.731	0.545	0.65	0.847	0.179	0.816	0.823	0.961	0.838

*P-values in bold are statistically significant*.

*Two-sided Wilcoxon–Mann–Whitney (continuous variables) or Chi-square exact test (categorical variable) comparing tumors with high vs. low expression.

Next, using univariate Cox regressions we evaluated the clinical relevance of clock core genes in metastatic melanoma. Among all nine genes analyzed, only *BMAL1* showed a significant prognostic value: high *BMAL1* expression was associated with longer overall survival (HR = 0.678, *P* = 0.002; Figures 1C,D). Importantly, multivariate Cox regression adjusting for age, gender, tumor pathologic stage, ulceration status, mitotic count, and Breslow thickness revealed *BMAL1* expression as an independent prognostic factor (Table 2). Additionally, the prognostic value of *BMAL1* expression in metastatic melanomas was confirmed in two other independent datasets (GSE6590 and GSE54467; Figures S1A,B and Table S2 in Supplementary Material).

**Table 2:** Multivariate Cox regression analysis of survival in The Cancer Genome Atlas metastatic melanomas.

	Overall survival	
Variables	HR (95%CI)	*P*-value
Age	1.024 (1.007–1.041)	**0.006**
**Gender**		
Male vs. female	1.158 (0.655–2.051)	0.612
**Pathologic stage**		
lll-IV vs. I–II	2.405 (1.427–4.053)	**<0.001**
**Ulceration status**		
Present vs. absent	0.994 (0.556–1.769)	0.985
Mitotic count	1.015 (0.986–1.045)	0.301
Breslow thickness	1.080 (1.004–1.161)	**0.038**
*BMAL1* expression	0.525 (0.369–0.746)	**<0.001**

*P-values in bold are statistically significant*.

*HR, hazard ratio; CI, confidence interval*.

### *BMAL1* Expression and the Overall Biological Profile of Metastatic Melanoma

**Figure 2:**
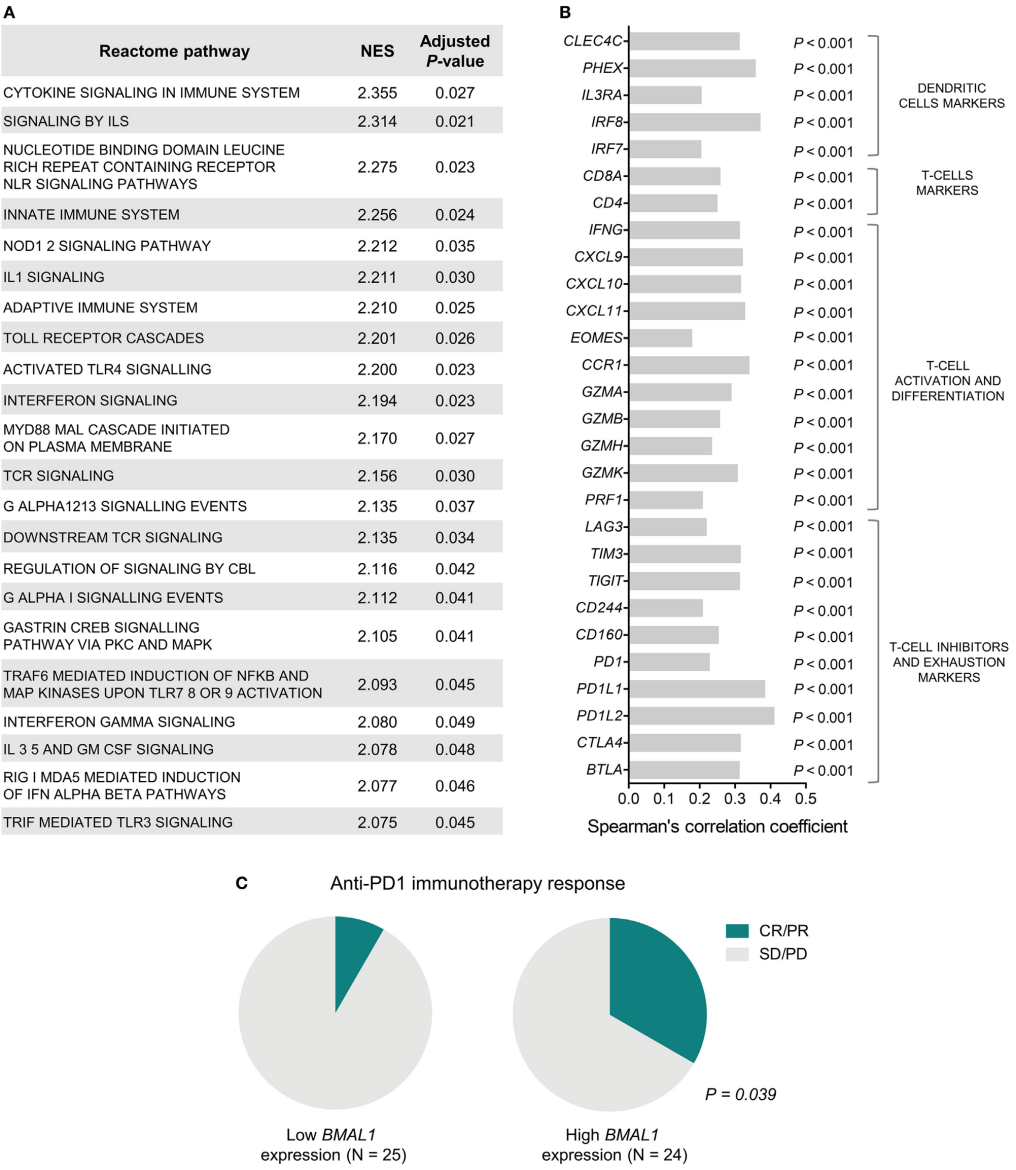
Aryl hydrocarbon receptor nuclear translocator-like protein 1 (*BMAL1*) expression positively correlates with antitumor immunity in metastatic melanomas. **(A)** Gene Set Enrichment Analysis (GSEA) using genes ranked according to the Spearman’s correlation coefficient between their expression and the expression of *BMAL1* in The Cancer Genome Atlas (TCGA) metastatic melanomas (*n* = 340). Normalized enrichment scores (NES) and *P-*values corrected by false discovery rate (FDR) were calculated using GSEA v3.0 and Reactome pathways. Only significantly enriched pathways (adjusted *P* < 0.05) are shown. **(B)** Spearman’s correlation coefficient between the expression of *BMAL1* and immune cells markers in TCGA metastatic melanomas. *P-*values were corrected by FDR. **(C)** Association between *BMAL1* expression (pretreatment biopsies) and clinical benefit of melanoma patients from anti-PD1 immunotherapy (nivolumab). RNAseq data and treatment response information were obtained from Ref. (42). Comparisons were performed using the Chi-square test. CR, complete response; PR, partial response; SD, stable disease; PD, progressive disease.

To investigate the biological mechanisms that likely underlie the impact of *BMAL1* expression on patient survival, we performed GSEA using genes ranked according to their Spearman’s correlation with *BMAL1* expression. Significantly enriched pathways presented positive NES and were mainly involved in the activation of the immune system (Figure 2A). In fact, in metastatic melanomas, *BMAL1* expression exhibited a strong positive correlation with the expression of dendritic cell markers, T-cell markers *CD4* and *CD8A*, and T-cell activation/differentiation markers (Figure 2B). This robust intratumoral activation of leukocytes was accompanied by the expression of T-cells exhaustion markers (Figure 2B), such as *CTLA4, PD1*, and *PDL1*, corroborating the fact that T-cell were chronically exposed to antigens (52, 53). Accordingly, patients with high *BMAL1* expression in pretreatment biopsies demonstrated improved response to anti-PD1 immunotherapy in comparison to patients expressing low *BMAL1* levels (Figure 2C).

The correlation between *BMAL1* expression and antitumor immune response was also confirmed in two additional independent datasets (GSE6590 and GSE54467; Figures S1C,D in Supplementary Material). Importantly, the expression of *BMAL1* was a prognostic factor independent of the percentage of leukocyte, monocyte, and neutrophil infiltration in TCGA melanomas (Table 3).

**Table 3:** Multivariate Cox regression analysis of overall survival in metastatic melanomas adjusted for the percentage of immune cell infiltration in The Cancer Genome Atlas metastatic melanomas.

	Overall survival	
Variables	HR (95% CI)	*P*-value
% Lymphocyte infiltration	0.975 (0.932–1.019)	0.261
% Monocyte infiltration	1.001 (0.902–1.111)	0.974
% Neutrophil infiltration	0.956 (0.673–1.357)	0.901
*BMAL1* expression	0.685 (0.550–0.854)	**<0.001**

*P-values in bold are statistically significant*.

*HR, hazard ratio; CI, confidence interval*.

### *BMAL1* Expression and the Mutational Load in Metastatic Melanomas

**Figure 3:**
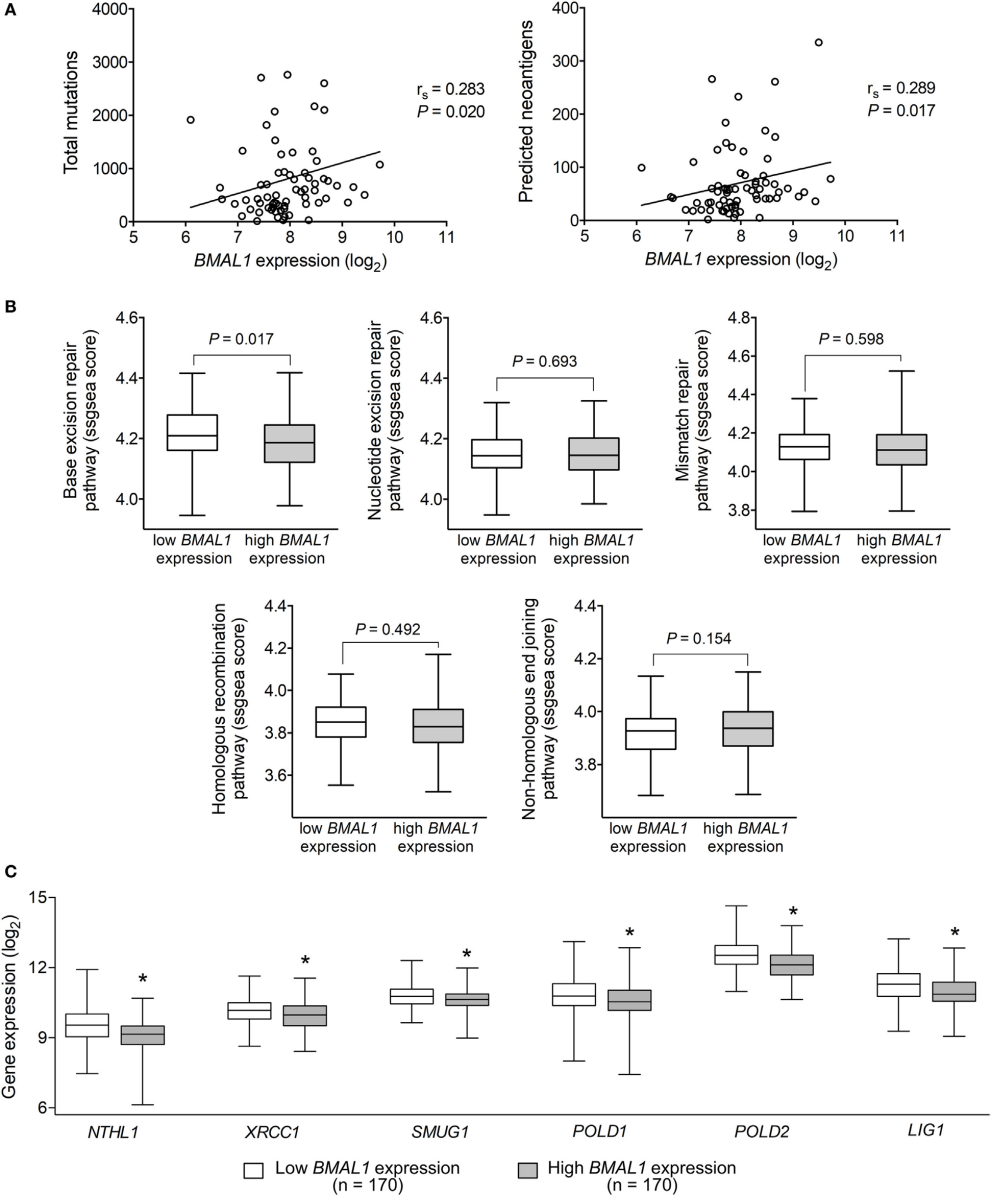
Aryl hydrocarbon receptor nuclear translocator-like protein 1 (*BMAL1*) expression positively correlates with the mutational and neoantigen load in metastatic melanomas. **(A)** Spearman’s correlation between the expression of *BMAL1* and the number of total mutations and predicted neoantigens in The Cancer Genome Atlas (TCGA) metastatic melanomas (*n* = 68). NetMHCpan-predicted neoantigens were obtained from Ref. (40). Gene expression of **(B)** DNA-repair programs and **(C)** selected base excision repair enzymes according to the expression of *BMAL1* in TCGA metastatic melanomas (*n* = 340). Pathway scores were calculated using single sample Gene Set Enrichment Analysis available in the GSVA R package. The median expression of *BMAL1* was used as the cutoff to dichotomize the population. The boxes extend from the 25th to the 75th percentile, the central bold line shows the median, and whiskers are drawn from minimum to maximum values. Comparisons were performed using the two-sided Wilcoxon–Mann–Whitney test. *Significantly different from the low *BMAL1* group.

Tumor somatic mutations can generate major histocompatibility complex Class I-associated neoantigens expression that plays a central role in inducing T-cell meditated antitumor cytolytic activity (54, 55). Interestingly, in metastatic melanomas, *BMAL1* expression positively correlated with the number of total somatic mutations and predicted neoantigens (Figure 3A). With that in mind, we investigated whether the expression of *BMAL1* was associated with the activation of different DNA-repair pathways. Using ssGSEA, we demonstrated that base excision repair is likely impaired in tumors expressing high *BMAL1* (Figure 3B). No significant differences were observed regarding the nucleotide excision repair, mismatch repair, homologous recombination, and non-homologous end joining DNA-repairing mechanisms. Importantly, the expression of base excision repair-related genes, such as *NTHL1, XRCC1*, and *SMUG1*, and the expression of general DNA repair-related genes, such as *POLD1, POLD2*, and *LIG1*, were downregulated in tumors expressing high *BMAL1* in all three datasets analyzed (Figure 3C; Figure S2 in Supplementary Material). High *BMAL1* expression was also associated with impaired DNA-repair capacity in human melanoma cell lines from the Cancer Cell Line Encyclopedia (Figure S3 in Supplementary Material).

## Discussion

Cancer onset, development, and progression have been linked to circadian disruption (17–19); however, the complex role of the tumor-autonomous molecular clock within these processes is yet poorly understood. Here, confirming previous reports in humans and in mice (32, 33, 35, 37), we showed that the expression of core components of the molecular clock machinery is severely repressed in melanomas. Moreover, we demonstrated that, for such tumors, high mRNA levels of *BMAL1* are associated with decreased gene expression of base excision repair enzymes and increased mutation load and predicted neoantigen presentation. The high incidence of antigenic peptides observed in metastatic melanomas with high *BMAL1* expression was accompanied by increased expression of cytotoxic T-cell activity markers in the tumor bulk and better prognosis. Even though our data do not provide a detailed mechanistic perspective, the present findings strongly support a role for *BMAL1* as a clinically relevant biomarker of DNA damage repair deficiency and intratumoral T-cell response. Thus, confirming such findings using common molecular techniques would be of great relevance for prognosis prediction and proposition of personalized therapeutic strategies.

Accumulating evidence implicates cell autonomous-circadian clocks in cancer development, as the disruption of peripheral systems of timekeeping seems to be a common event in malignant tissues (17, 18). As demonstrated here for metastatic melanomas, the expression of most clock core genes is downregulated in several types of cancers when compared to normal tissue (28, 32, 56–61). Moreover, the overexpression of *PER1* and *PER2* has been shown to impair tumor proliferation and induce apoptosis in lung, prostate, and pancreatic cancer (29, 62, 63), reinforcing the idea that the molecular clock machinery may be considered as a new therapeutic target.

The protein encoded by *BMAL1* belongs to the family of the bHLH-PAS structural domain transcription factors and it is estimated to control the expression of more than 150 target genes, including the clock genes *CRY1, CRY2, NR1D1, PER1, PER2*, and *PER3* (64). *BMAL1* has also been revealed as a candidate gene for susceptibility to hypertension, diabetes, and obesity, and mutations in *BMAL1* have been linked to infertility and metabolic dysfunctions (65–70). Here, we demonstrated that, in metastatic melanomas, the expression of *BMAL1* is a robust positive prognostic factor of overall survival and has a negative association with the expression of key DNA-repair enzymes, such as *POLD1, POLD2*, and *LIG1*. Accordingly, in colorectal cancer, downregulation of *BMAL1* gene expression accelerates cell proliferation *in vitro*, promotes tumor growth in mice, and decreases DNA damage induced by cisplatin (71). Moreover, high *BMAL1* expression is associated with increased sensitivity of colorectal cancer cells to oxaliplatin *in vitro* and *in vivo*, and predicts favorable outcome for patients treated with oxaliplatin-based chemotherapy (72). *BMAL1* expression also positively correlates with patient survival in pancreatic ductal adenocarcinomas (61), causes growth inhibition in lymphoma/leukemia cells (58), negatively impacts DNA-repair capacity of mice fibroblast (73), but promotes proliferation in malignant pleura mesothelioma (74), suggesting that its role in tumorigenesis is complex and tissue-specific.

Although it has been shown that alterations in the tumor molecular clock impact some parameters of tumor progression (28–30, 62, 63), the influence of endogenous oscillatory systems on the cellular composition of the tumor microenvironment is largely unknown. In this sense, our data indicate that the prolonged survival of metastatic melanoma patients with high *BMAL1* bulk expression is associated with a robust intratumoral T-cell infiltration/activation, which can be partially explained by the increased neoantigen load that likely reflects the impaired DNA-repair capacity. Previous reports have also linked DNA-repair deficiency to increased mutational load and antitumor immune response in melanomas, lung, colorectal, and endometrial cancers (75–78). It is now clear that DNA repair and genomic instability have a pivotal role in the modulation of antitumor immune responses (79); thus, understanding their interplay with tumor-autonomous clocks may provide clinically relevant insights.

Immunotherapies that boost the ability of T lymphocytes to combat tumor cells have demonstrated therapeutic efficiency in a variety of solid tumors. Monoclonal antibodies against T-cell checkpoint proteins, such as CTLA-4, PD-1, and PD-L1, have now been approved for melanoma treatment and are associated with robust durable responses, but only in a subset of tumors (80–82). Thus, there is a need to identify biomarkers that will allow the selection of treatment-responsive patients, avoid unnecessary toxicity, and help personalize therapy regimens (83). Metastatic melanomas presenting high *BMAL1* expression have impaired DNA-repair capacity combined with increased mutation/neoantigen load, T-cell intratumoral infiltration, and T-cell expression of exhaustion markers, all of which have been shown to predict good clinical response to the treatment with immune checkpoint inhibitors (78, 84–87). In fact, we showed that high *BMAL1* expression in pretreatment melanoma samples is associated with clinical benefit from anti-PD1 immunotherapy. Considering that whole-genome and -transcriptome sequencing is expensive and time-consuming, profiling a smaller fraction of genes could serve as a useful tool to help translate those findings into routine clinical practices (88). Therefore, the present data indicate that *BMAL1* expression in melanoma patients must be considered as a relevant marker for immunotherapy efficacy. Nevertheless, larger clinical studies are necessary to validate the potential of *BMAL1* alone, or along with other biomarkers, in discriminating responders from non-responders in immunotherapy regiments.

## Conclusion

The molecular characterization of melanomas using high-throughput approaches has the potential to generate insights into their biological heterogeneity, having important implications for prognosis and therapy. In this sense, our data highlight the relevance of further studies focusing on the biological and clinical relevance of the tumor-autonomous molecular clock machinery. Overall, we demonstrated that, in metastatic melanoma, a high bulk *BMAL1* expression seems to be associated with a “too tumorigenic” program and could be a marker for immunotherapy response.

## Ethics Statement

All data presented in this manuscript are public and freely available. We did not perform any human or animal related experiments. All analyses and conclusions were drawn from the following public datasets: The Cancer Genome Atlas (TCGA), Genotype-Tissue Expression (GTEx), Gene Expression Omnibus, and datasets from Ref. (40, 42). In all mentioned papers, the authors stated that all procedures were carried out according ethical rules.

## Author Contributions

LA and GK designed the study, analyzed the data, and drafted the manuscript. All authors provided insightful discussion during data acquisition and aided in the writing process of the manuscript. All authors critically revised the manuscript. All authors have approved the definitive version of the manuscript and agreed to be accountable for all aspects of the study in ensuring that questions related to the accuracy or integrity of any part of the study are appropriately investigated and resolved. All persons designated as authors qualify for authorship, and all those who qualify for authorship are listed.

## Conflict of Interest Statement

All authors state no conflict of interest that could have impacted the development of this study.
