# Supplementary material for: Expression of the Circadian Clock Gene BMAL1 Positively Correlates With Antitumor Immunity and Patient Survival in Metastatic Melanoma
Source: Front Oncol. 2018 Jun 12;8:185. doi: 10.3389/fonc.2018.00185 (PMC6005821; doi:10.3389/fonc.2018.00185)
Supplement: Supplementary file 1 [file data_sheet_1.PDF]

Expression of the circadian clock gene *BMAL1* positively correlates with antitumor immunity and patient survival in metastatic melanoma

Leonardo Vinícius Monteiro de Assis[1#], Gabriela Sarti Kinker[2#], Maria Nathália Moraes[1], Regina P. Markus[2], Pedro Augusto Fernandes[2], Ana Maria de Lauro Castrucci[1,3]

de Assis, L.V.; Kinker, G.S; Moraes, M.N; Markus, R.P.; Fernandes, P.A; Castrucci, A.M.L.

[1)] Laboratory of Comparative Physiology of Pigmentation, Department of Physiology, Institute of Biosciences, University of São Paulo, São Paulo, Brazil.
[2)] Laboratory of Neuroimmunemodulation, Department of Physiology, Institute of Biosciences, University of São Paulo, São Paulo, Brazil
[3)] Department of Biology, University of Virginia, Charlottesville, VA, USA

*Corresponding author: Leonardo V.M. de Assis or Gabriela S. Kinker, Department of Physiology, Institute of Biosciences, University of Sao Paulo, R. do Matão, trav. 14, no. 101, São Paulo, 05508-900, Brazil; e-mail: deassis.leonardo@usp.br or gabriela.kinker@usp.br
[#] these authors contributed equally to this study.

Running title: *BMAL1* correlates with patient survival in metastatic melanoma

SUPPORTING INFORMATION

Supporting Materials and Methods

Gene expression validation datasets

Additional metastatic melanoma gene expression datasets (GSE65904, n = 176 and GSE54467, n = 71) were retrieved from the public repository Gene Expression Omnibus using GEOquery and Biobase Bioconductor R packages (http://www.bioconductor.org/). GSE65904 data were generated using Illumina HumanHT-12 V4.0 Expression Beadchip arrays, processed according to the cubic spline and quantile normalization method and log_2-transformed (Cirenajwis et al., 2015). GSE54467 data were generated using Sentrix Human-6 v3 Expression BeadChip arrays, processed according to the variance-stabilizing transform (VST) and quantile normalization method and log_2 transformed (Jayawardana et al., 2015). Gene expression data from 49 different human melanoma cell lines were obtained from the Cancer Cell Line Encyclopedia website – Broad Institute (CCLEC and GDSCC, 2015). Data were generated using the Illumina HiSeq 2000/2500 RNA sequencing platform, counted using htseq2, upper quartile normalized and $log_2(x+1)$ transformed.

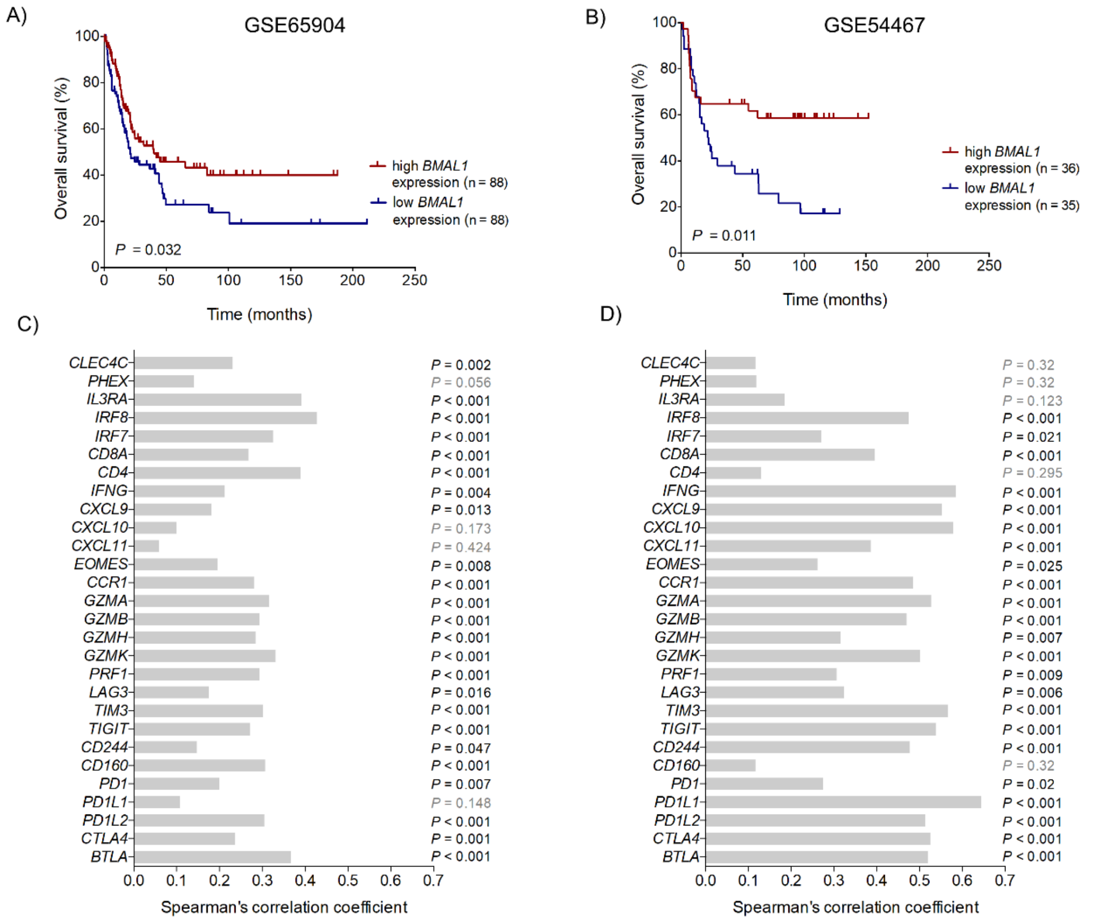

Supplementary Figure 1. *BMAL1* expression positively correlates with antitumor immune response and patient survival in two independent datasets. Analysis of two independent expression datasets of metastatic melanomas available in the GEO repository (GSE65904 and GSE54467). A-B) Kaplan-Meier survival according to the expression of *BMAL1* (median used as the cutoff). Comparisons were performed using the log-rank test. C-D) Spearman's correlation coefficient between the expression of *BMAL1* and immune cell markers.

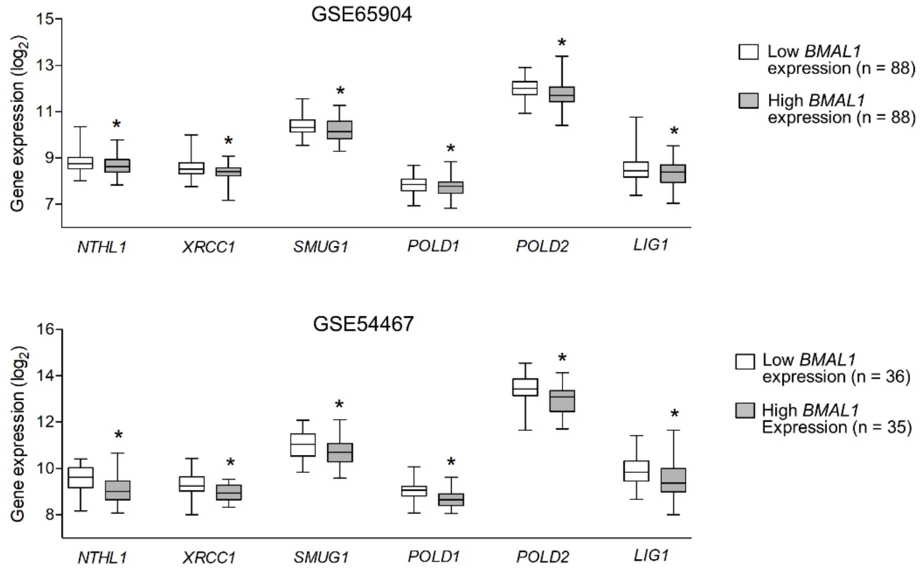

Supplementary Figure 2. High *BMAL1* expression is associated with impaired DNA repair capacity in two independent datasets. Analysis of two independent expression datasets of metastatic melanomas available in the GEO repository (GSE65904 and GSE54467). The expression of base excision repair enzymes according to the expression of *BMAL1* (median used as the cutoff). The boxes extend from the 25th to the 75th percentile, the central bold line shows the median, and whiskers are drawn from minimum to maximum. Comparisons were performed using the two-sided Wilcoxon-Mann-Whitney test. * Significantly different from the low *BMAL1* group.

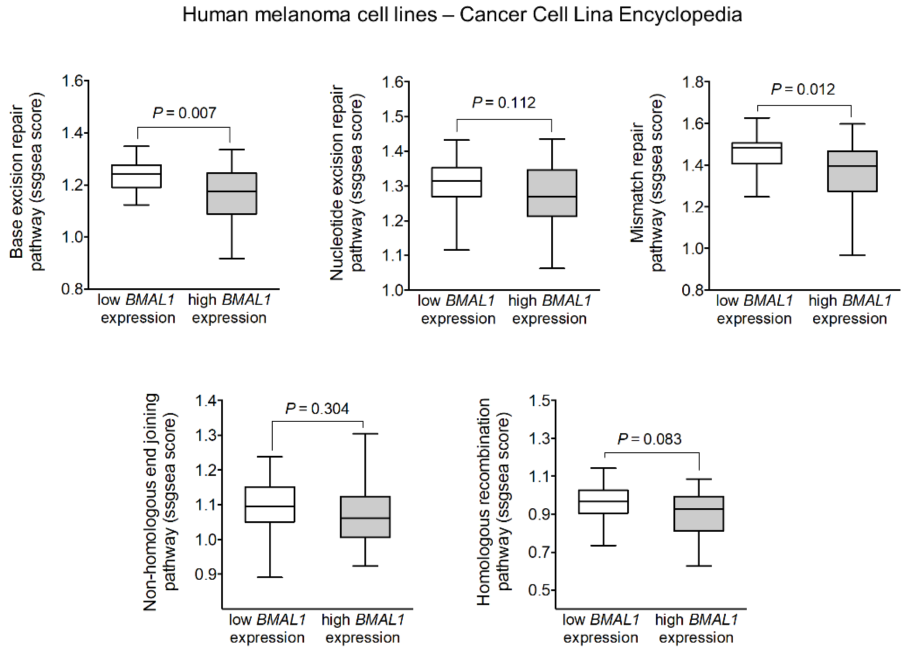

Supplementary Figure 3. Impact of *BMAL1* expression on the DNA repair capacity of 49 human melanoma cell lines (CCLE – Broad Institute). Pathway scores were calculated using single sample Gene Set Enrichment Analysis (ssGSEA) available in the GSVA R package. The median expression of *BMAL1* was used as the cutoff to dichotomize the population. The boxes extend from the 25th to the 75th percentile, the central bold line shows the median, and whiskers are drawn from minimum to maximum values. Comparisons were performed using the two-sided Wilcoxon-Mann-Whitney test.

Supplementary Table 1 – Association between clinicopathological features and the expression of *NR1D1*, *PER2*, and *PER3* in metastatic melanomas. P-values in bold are significant.

	***NR1D1* expression**			***PER2* expression**			***PER3* expression**		
**Variables**	**Low**	**High**	***P*-value***	**Low**	**High**	***P*-value***	**Low**	**High**	***P*-value***
Age, y									
Mean (SD)	54.2 (16.6)	57.5 (15)	0.057	55.6 (16.4)	56.1 (15.5)	0.754	53.2 (16.2)	58.4 (15.2)	**0.002**
Gender, %									
Male	45	55	**0.015**	44	56	**0.028**	40	60	**<0.001**
Female	59	41		56	44		64	36	
Pathologic stage, %									
I-II	46	54	0.247	44	56	0.132	43	57	**0.037**
III-IV	53	47		53	47		55	45	
Ulceration status, %									
Absent	52	48	1	46	54	0.267	46	54	0.889
Present	52	48		54	46		45	55	
Mitotic count, mitosis/mm^2									
Mean (SD)	7 (7.7)	6.2 (6)	0.465	7.2 (7.4)	6 (6.3)	0.276	6.3 (7.1)	6.8 (6.8)	0.613
Breslow thickness, mm									
Mean (SD)	3 (3.3)	3.8 (5.8)	0.179	3.4 (3.3)	3.5 (5.8)	0.823	3.4 (5.5)	3.4 (3.8)	0.961

*Two-sided Wilcoxon-Mann-Whitney test (continuous variables) or chi-squared test (categorical variables).

Supplementary Table 2 – Multivariate Cox regression analysis of overall survival in metastatic melanomas (validation datasets). P-values in bold are significant.

	**Overall survival**			
	**GSE65904**		**GSE54467**	
**Variables**	**HR (95% CI)**	***P*-value**	**HR (95% CI)**	***P*-value**
Age	1.004 (0.990 – 1.019)	0.559	1.026 (1.003 – 1.050)	**0.023**
Gender				
Male vs. Female	1.204 (0.796 – 1.822)	0.378	0.999 (0.524 – 1.904)	0.998
***BMAL1* expression**	0.550 (0.309 – 0.977)	**0.041**	0.310 (0.132 - 0.727)	**0.007**

HR, hazard ratio; CI, confidence interval.

REFERENCES

Cclec, and Gdscc (2015). Pharmacogenomic agreement between two cancer cell line data sets. *Nature* 528**,** 84-87.

Cirenajwis, H., Ekedahl, H., Lauss, M., Harbst, K., Carneiro, A., Enoksson, J., Rosengren, F., Werner-Hartman, L., Torngren, T., Kvist, A., Fredlund, E., Bendahl, P.O., Jirstrom, K., Lundgren, L., Howlin, J., Borg, A., Gruvberger-Saal, S.K., Saal, L.H., Nielsen, K., Ringner, M., Tsao, H., Olsson, H., Ingvar, C., Staaf, J., and Jonsson, G. (2015). Molecular stratification of metastatic melanoma using gene expression profiling: Prediction of survival outcome and benefit from molecular targeted therapy. *Oncotarget* 6**,** 12297-12309.

Jayawardana, K., Schramm, S.J., Haydu, L., Thompson, J.F., Scolyer, R.A., Mann, G.J., Muller, S., and Yang, J.Y. (2015). Determination of prognosis in metastatic melanoma through integration of clinico-pathologic, mutation, mRNA, microRNA, and protein information. *Int J Cancer* 136**,** 863-874.
